# Antibacterial Activity of a Lytic Enzyme Encoded by *Pseudomonas aeruginosa* Double Stranded RNA Bacteriophage phiYY

**DOI:** 10.3389/fmicb.2018.01778

**Published:** 2018-08-03

**Authors:** Yuhui Yang, Shuai Le, Wei Shen, Qian Chen, Youying Huang, Shuguang Lu, Yinling Tan, Ming Li, Fuquan Hu, Yang Li

**Affiliations:** ^1^Department of Microbiology, Army Medical University, Chongqing, China; ^2^Department of Medical Laboratory, Chengdu Military General Hospital, Chengdu, China; ^3^Biomedical Analysis Center, Army Medical University, Chongqing, China; ^4^Trauma Center of PLA, State Key Laboratory of Trauma, Burns and Combined Injury, Institute of Surgery Research, Daping Hospital, Army Medical University, Chongqing, China

**Keywords:** dsRNA bacteriophage, *Pseudomonas aeruginosa*, lysin, VAPGH, antibacterial activity

## Abstract

Multidrug-resistant *Pseudomonas aeruginosa* is one of the most life-threatening pathogens for global health. In this regard, phage encoded lytic proteins, including endolysins and virion-associated peptidoglycan hydrolases (VAPGH), have been proposed as promising antimicrobial agents to treat *P. aeruginosa*. Most dsDNA phages use VAPGH to degrade peptidoglycan (PG) during infection, and endolysin to lyse the host cells at the end of lytic cycle. By contrast, dsRNA phage encodes only one lytic protein, which is located in the viral membrane to digest the PG during penetration, and also serves as an endolysin to release the phage. Currently, there are only seven sequenced dsRNA phages, and phiYY is the only one that infects human pathogen *P. aeruginosa.* In this study, dsRNA phage phiYY encoded lysin, named Ply17, was cloned and purified. Ply17 contains a PG-binding domain and a lysozyme-like-family domain. Ply17 exhibited a broad antibacterial activity against the outer membrane permeabilizer treated Gram-negative bacteria. The best lytic activity was achieved at 37°C, pH 7.5, in the presence of 0.5 mM EDTA. Moreover, it could effectively lyse Gram-positive bacteria directly, including *Staphylococcus aureus*. Therefore, dsRNA phage encoded Ply17 might be a promising new agent for treating multidrug-resistant pathogens.

## Introduction

*Pseudomonas aeruginosa* is one of the most life-threatening opportunistic pathogens that cause infections of bloodstream, urinary tract, burn wound, as well as airway of cystic fibrosis patient (Jonckheere et al., 2018; Waters and Grimwood, 2018). Moreover, the emergence and increase of multidrug resistance in clinical isolates are worrying (Alessa et al., 2018). Thus, alternative treatments are needed to cope with this problem, and bacteriophage (phage) and phage derived lysin are promising antimicrobial agents (Lood et al., 2015; Forti et al., 2018; Gutierrez et al., 2018).

Phages are viruses that specifically infect bacteria (Salmond and Fineran, 2015). There are two major weapons utilized by phage to break the bacterial cell wall. In the initial step of infection, phage uses virion-associated peptidoglycan hydrolases (VAPGH) to slightly degrade peptidoglycan (PG) to inject the phage genetic material into the cell (Rodriguez-Rubio et al., 2013; Latka et al., 2017). At the end of infection cycle, phage encoded holin forms pores in the inner membrane, and followed by access of endolysin to the cell wall where it degrades PG (Gerstmans et al., 2017). Then cell lysed and the mature phage particle released. Thus, endolysin and VAPGH are promising alternative antimicrobial agents, and there are renewed interests in studying and exploiting their potentials in combating pathogens (Defraine et al., 2016; Sao-Jose, 2018).

Endolysin has been extensively studied in the last decade. Most phage endolysin possess a cell wall binding domain (CBD) and one or two catalytic domains, which can be classified into six different types according to the cleaving activity against PG (Gutierrez et al., 2018). Endolysin has been shown to be active in killing pathogens (Becker et al., 2016), degrading biofilms (Gutierrez et al., 2014) and protecting animal models from pathogen infection (Lood et al., 2015).

Compared with endolysin, VAPGH is less studied, but its therapeutic value has attracted more attention recently (Rodriguez-Rubio et al., 2013; Latka et al., 2017). To inject genomes into bacterial cytoplasm, phages have to degrade the PG through VAPGH, which has been classified into three categories base on the cleaving site on PG, including glycosidases, amidases, and endopeptidases (Latka et al., 2017). Majority of the VAPGH are glycosidases, such as Gp16 from phage T7 (Moak and Molineux, 2000). The most common locations of VAPGH in the viron is phage tail and baseplate to facilitate the degradation of PG after phage adsorption (Briers et al., 2008). And for membrane-containing phages, VAPGH are located in the viral membrane, such as Gp5 in phage phi6 (Caldentey and Bamford, 1992). VAPGH has been shown to be active against Gram-positive bacteria, such as *Staphylococcus aureus* (Rashel et al., 2008; Rodriguez-Rubio et al., 2012), and can also kill outer membrane-permeabilized Gram-negative bacteria, such as *P. aeruginosa* (Lavigne et al., 2004).

Most DNA phages encode endolysin and VAPGH separately. Interestingly, dsRNA phages phi6 and phi13 encode only one lytic protein, which is located in the viral membrane to digest the PG during penetration, and also serves as an endolysin to release the phage (Caldentey and Bamford, 1992; Daugelavicius et al., 2005). Currently, only seven dsRNA phages have been sequenced, and phiYY is the only one that infects human pathogen *P. aeruginosa* (Yang et al., 2016), while other six dsRNA phages infect *Pseudomonas syringae* (Mantynen et al., 2018). In this study, the dsRNA phage phiYY encoded lysin, named Ply17, was purified and its antimicrobial activity was tested against a panel of pathogens.

## Materials and Methods

### Bacterial Strains and Culture Conditions

*Pseudomonas aeruginosa* PAO1 and lytic phage phiYY (Yang et al., 2016) were stored in our laboratory at -80°C in glycerol. *P. aeruginosa* clinical isolated strains (Sun et al., 2013), *Escherichia coli, S. aureus* (Zhang et al., 2014), and *Staphylococcus epidermidis* strains were routinely grown in Luria–Bertani (LB) broth with aeration at 37°C.

### Ply17 Protein Purification

The *Ply17* gene was amplified by PCR, using cDNA of phiYY genome as template. The PCR primers were Ply17-F: 5′-GGGAATTCCATATGGCTGCCCAGGGTCGC-3′ and Ply17-R: 5′-CGGAATTCTGCGAATAGAGCT-3′. The PCR product was purified and digested with *Nde*I/*Eco*RI. The digested PCR product was then ligated into *Nde*I/*Eco*RI-treated PET-28a to generate pET-Ply17, which was transferred into *E. coli* BL21(DE3) and selected on LB agar containing kanamycin (50 μg/ml).

To induce the expression of His-tagged Ply17, transformed *E. coli* strain was cultured in LB containing 50 μg/ml kanamycin until OD_600_ = 0.6. Then, 0.5 mM IPTG was used to induce the expression of Ply17. After 12 h induction at 37°C, cells were pelleted and the His-tagged Ply17 was purified through Ni-NTA column as previously described (Dong et al., 2015).

Purified protein was confirmed by SDS/PAGE stained with Coomassie Brilliant Blue and stored at -80°C after the buffer was changed to storage buffer (20 mM Tris–HCl, 100 mM NaCl, 10% glycerol, pH 7.5) by ultrafiltration.

### Determination of the Antimicrobial Activity

The antimicrobial activity of Ply17 was determined by colony forming units (CFU) reduction analysis as previously described (Rodriguez et al., 2011; Dong et al., 2015). For Gram-negative bacteria, early log phase cells were pelleted and resuspended in 20 mM Tris–HCl buffer (pH 7.5) supplemented with 0.1 M EDTA for 5 min at room temperature. The high concentration of EDTA is toxic to the cells, thus 5 min is applied to treat the outer membrane. Then, cells were pelleted and washed with 20 mM Tris–HCl buffer three times to remove the remaining EDTA. Next, 20 μl of Ply17 (5 mg/ml) was added into 80 μl of resuspended bacteria with a final concentration of Ply17 as 1 mg/ml. After 30 min incubation at 37°C, the mixture was serially diluted by 10-fold and plated on LB agar plates. Then, CFU was calculated after 24 h incubation to determine the viable cell number.

Factors affecting Ply17 function were analyzed using early log phase bacteria under different reaction conditions, including Ply17 concentration (0–1.5 mg/ml), pH (4.0–11.0).

To test the synergism between EDTA and Ply17, 0.1 M EDTA-treated PAO1 was mixed with 1 mg/ml Ply17 in the presence of different EDTA concentrations (0–5.0 mM) for 30 min at 37°C. Then, CFU was calculated after 24 h cultivation.

To test the thermal stability, Ply17 was incubated at different temperatures (30–90°C) for 10 min. Then, use the standard condition to test its lytic activity through CFU reduction analysis.

To test the lytic activity against Gram-positive bacteria, cells were pelleted and resuspended in 20 mM Tris–HCl buffer (pH 7.5) without EDTA. Then 20 μl of Ply17 (5 mg/ml) was added into 80 μl of resuspended bacteria. The remaining protocol is same as treating Gram-negative bacteria as described previously. All experiments were repeated three times. The values are the means and standard deviations from triplicate assays.

## Results

### Ply17 Sequence Analysis and Protein Purification

Genome annotation revealed a putative muramidase, named Ply17, in the phiYY genome segment S (NCBI: KX074203.1). Ply17 is also detected in the viral particles by HPLC-MS after SDS-PAGE, indicating that Ply17 is viron associated (Yang et al., 2016).

**FIGURE 1:**
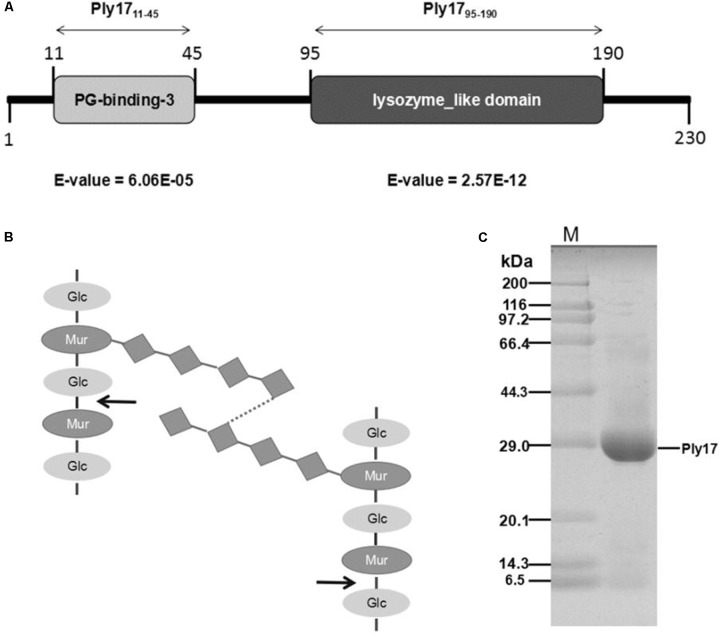
Ply17 modular architecture and purification. **(A)** Schematic representation of the domain organization of Ply17. Conserved cell wall binding domains and catalytic domain are annotated and displayed as light gray and dark gray boxes, respectively. **(B)** The structure of peptidoglycan is shown, and Ply17 is predicted to digest the beta-1,4-linked polysaccharides. The digestion site is indicated with an arrow. **(C)** Purified Ply17 protein is observed in SDS-PAGE (15%) stained with Coomassie Brilliant Blue.

*In silico* analysis of Ply17 predicted a 230-amino acid protein (25.2 kDa) with two independent domains: the PG-binding domain and lysozyme-like-family domain (**Figure 1A**). The PG-binding domain is responsible for cell wall binding, which is present in most endolysins, but absented in VAPGHs. The lysozyme-like-family domain is similar to muramidase of dsRNA phage phi13, with a predicted function of hydrolysis of beta-1,4-linked polysaccharides (**Figure 1B**).

Ply17 with a His-tag at the C-terminus was purified from *E. coli* cells bearing an IPTG-inducible expression plasmid pET-Ply17. The purified protein band was observed with correct mass (25.2 kDa), as was determined by SDS/PAGE stained with Coomassie Brilliant Blue (**Figure 1C**).

### Lytic Activity of Ply17

**FIGURE 2:**
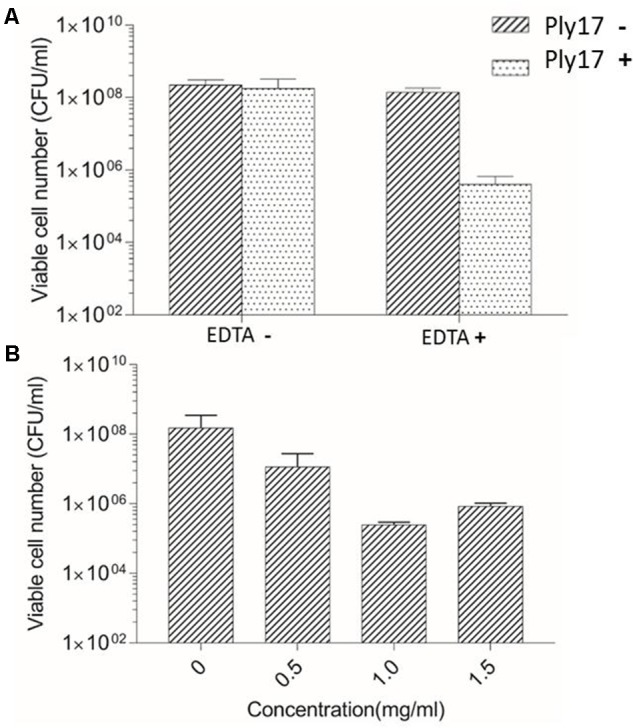
Lytic activity of Ply17 against PAO1. **(A)** Ply17 (1 mg/ml) significantly reduced the EDTA-treated viable bacteria more than 2 logs, while Ply17 cannot kill PAO1 directly. **(B)** Lytic activity of different concentrations of Ply17 against PAO1. All experiments were repeated three times. The values are the means and standard deviations from triplicate assays.

The antibacterial activity of Ply17 was tested by CFUs reduction assay (Dong et al., 2015), using *P. aeruginosa* strain PAO1 as target. Gram-negative bacterium is rarely sensitive to endolysin due to the outer membrane barrier. Thus, 0.1 M EDTA was used as outer membrane permeabilizer to treat PAO1. Our data showed that, in the presence of permeabilizer, Ply17 induced PAO1 lysis and the viable cell significantly decreased after 30 min treatment, while no killing was observed in the absence of EDTA (**Figure 2A**). Meanwhile, different concentrations of Ply17 were tested. The addition of 500 μg/ml Ply17 reduced the viable numbers of EDTA-treated PAO1 by 1 log unit, while the most significant reduction was observed when 1 mg/ml Ply17 was used (**Figure 2B**). These data further confirmed the antimicrobial activity of Ply17.

### Influence of EDTA, Temperature, and pH on Ply17 Antibacterial Activity

**FIGURE 3:**
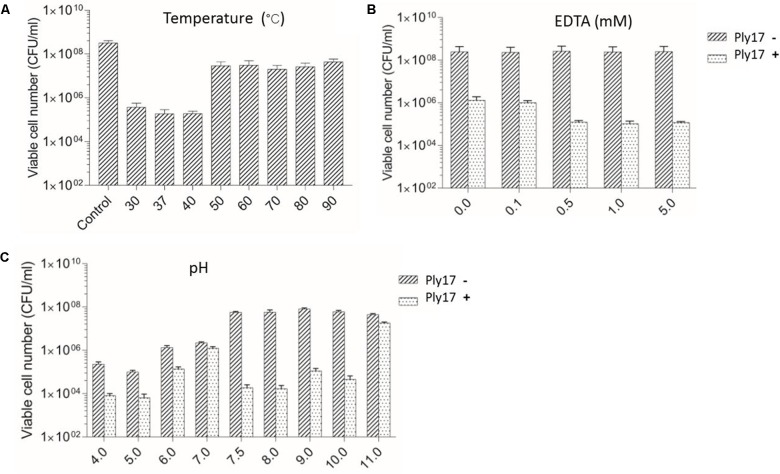
Lytic activity of Ply17 against PAO1 under different conditions. The antimicrobial activity of Ply17 on 0.1 M EDTA-treated PAO1 was performed at different temperatures **(A)**, EDTA concentrations **(B)**, and pH values **(C)**. EDTA displays a synergistic effect with Ply17 in killing PAO1 **(B)**. The best lytic activity was observed at 37°C, pH 7.5, in the presence of 0.5 mM EDTA.

To test the thermal stability of lysin, Ply17 was incubated under different temperatures for 10 min, then cooled to room temperature and used to test its antimicrobial activity under 37°C. The results showed that Ply17 is stable under 40°C, but its activity significantly decreased after heat-treatment at 50°C (**Figure 3A**).

Meanwhile, EDTA has a synergistic effect with Ply17 in killing PAO1. As the concentration of EDTA in the reaction buffer increased to 0.5 mM, the antibacterial activity of Ply17 increased accordingly, but plateaued after that concentration (**Figure 3B**).

The low pH values of the reaction buffer significantly affect cell viability, while the relative high lytic activity was observed at pH value of 7.5–8, with a reduction of 3–4 log units of viable cells compared to no Ply17 addition control (**Figure 3C**). The isoelectric point of Ply17 is predicted to be 7.0. Thus, its activity is significantly impaired at pH 7.0.

### The Broad Bactericidal Activity of Ply17

**FIGURE 4:**
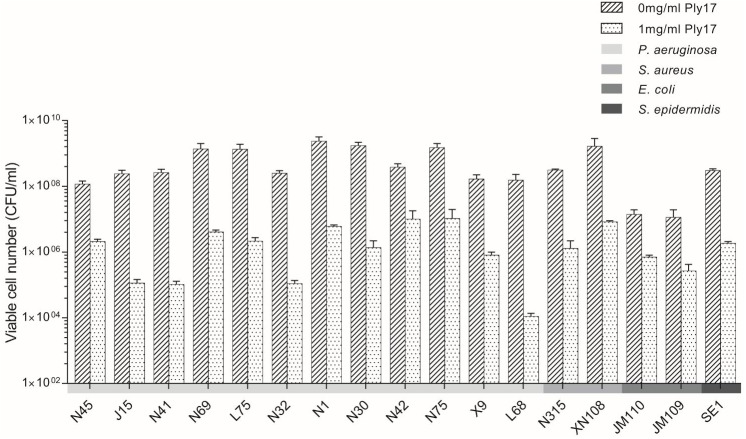
The bactericidal activity of Ply17 versus Gram-negative and Gram-positive bacteria. Ply17 has a broad antimicrobial spectrum, which is active against *P. aeruginosa, E. coli, S. aureus*, and *S. epidermidis*.

The activity spectrum of Ply17 against different *P. aeruginosa* strains and other bacterial species is shown in **Figure 4**. The viabilities of the 12 clinical isolates of *P. aeruginosa* (Sun et al., 2013) and two *E. coli* strains (JM110 and JM109) reduced 1–4 log unit following the incubation with Ply17 for 30 min.

**FIGURE 5:**
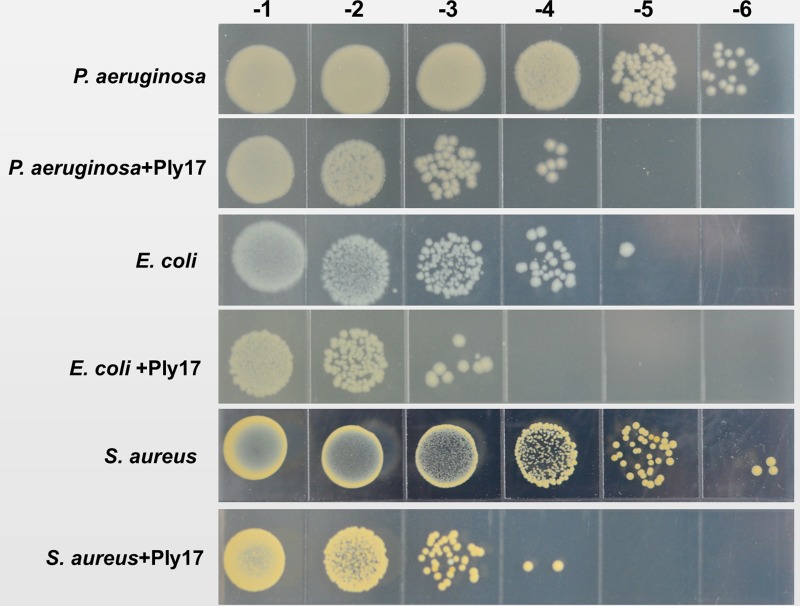
Representative pictures of cell viability before and after Ply17 treatment. To measure viability, 10-fold dilutions of log phase bacteria and Ply17-treated cells were spotted onto agar plates. Representative pictures of *P. aeruginosa* PAO1, *E. coli* JM109, and *S. aureus* XN108 showed that Ply17 reduced the viable cell by ∼2 log units.

To test whether Ply17 is active against Gram-positive bacteria, Ply17 was added to *S. aureus* strain (N315, XN108; Zhang et al., 2014) and *S. epidermidis* strain SE1 without EDTA treatment, because the PG of Gram-positive is exposed without outer membrane. As shown in **Figures 4**, **5**, Ply17 was able to lyse all three Gram-positive strains and reduced the viable cells by 2 log units. Representative pictures of cell viability before and after Ply17 treatment are shown in **Figure 5**.

## Discussion

In this study, we reported the antibacterial activity of a lytic enzyme Ply17 encoded by double stranded RNA bacteriophage phiYY (Yang et al., 2016). Lysin is usually classified into endolysin and VAPGH, based on their different functions in the phage infection cycle (Gutierrez et al., 2018). However, lysin encoded by dsRNA phage is quite unique. It is located in the viron membrane to degrade the PG during the initial infection process, and also serves as endolysin at the final step of phage infection cycle (Daugelavicius et al., 2005). Currently, lysin P5 from phi6 is the only lysin isolated from dsRNA phage, and it was purified from disrupted viral particles (Caldentey and Bamford, 1992). In this study, we successfully purified phiYY encoded lysin Ply17 with a His-tag at the C-terminus from *E. coli* cells bearing an IPTG-inducible expression plasmid pETPly17.

The antimicrobial activity of Ply17 was confirmed by its ability to reduce the viability of *P. aeruginosa* PAO1 (**Figure 2**). However, all the Gram-negative bacteria possess outer membrane to prevent the entry of lysin into the cell wall (Nikaido, 2003), thus Ply17 cannot kill *P. aeruginosa* directly (**Figure 2A**). Our data showed that, when pre-treated with EDTA, a chelator which disrupts the outer membrane by removing the stabilizing cations (Oliveira et al., 2014; Dong et al., 2015), Ply17 significantly reduced the cell viability, confirming its antimicrobial activity.

Lysin Ply17 showed a broad lytic activity against both Gram-positive and Gram-negative species (**Figure 4**), including *P. aeruginosa, E. coli, S. aureus*, and *S. epidermidis.* In contrast, dsRNA phage phi6 encoded lysin P5 only lyses Gram-negative bacteria, but not Gram-positive bacteria (Caldentey and Bamford, 1992). One possible reason could be due to their differences in the cleaving site specificity. Lysin P5 from phage phi6 acts on the peptide component of PG, which is quite diverse in Gram-positive bacteria (Alcorlo et al., 2017). On the contrary, lysin Ply17 was predicted to contain a lysozyme-like-family domain, which cleaves the beta-1,4-linked polysaccharides. The polysaccharides chain is made up of *N*-acetylglucosamine and *N*-acetylmuramic acid residues linked by beta-1,4 glycosidic bonds, which is very conservative (Pazos et al., 2017). Thus, Ply17 can directly cleaves the PG of Gram-positive bacteria without EDTA treatment (**Figure 5**).

To target Gram-negative bacteria infection, lysin must pass through the outer membrane. Some lysins possess this capability. For example, *Acinetobacter baumannii* phage lysin PlyF307 is active against all clinical strains of *A. baumannii*, and the killing is outer membrane permeabilizer independent (Lood et al., 2015). The molecular mechanism is yet to be determined. But the C-terminal region of PlyF307 contains a positively charged outer membrane-destabilizing domain, which might play a role. Moreover, engineered lysin can also penetrate the outer membrane (Sao-Jose, 2018). Artilysin is a novel engineered lysin, which consists of an endolysin and a specific lipopolysaccharide destabilizing peptide that can translocate the endolysin into cell wall (Gerstmans et al., 2016). Therefore, artilysin can directly kill both Gram-positive and Gram-negative bacteria with high efficiency (Defraine et al., 2016; Schirmeier et al., 2018). Thus, further engineering of Ply17 might improve its ability to kill Gram-negative bacteria and enhance its killing efficiency against Gram-positive pathogens.

## Author Contributions

YL and FH conceived the study. YY, SLe, and WS performed the experiments. SLu, YT, and ML analyzed the data. QC and YH provided intellectual support. SLe wrote the paper. All authors read and approved the final manuscript for publication.

## Conflict of Interest Statement

The authors declare that the research was conducted in the absence of any commercial or financial relationships that could be construed as a potential conflict of interest.
